# Meeting of the American Medical Association

**Published:** 1869-04

**Authors:** 


### Editorial Department.

### Meeting of the American Medical Association.

It will be remembered that the President-elect of the American Medical Association, in his address of acceptance, gave the idea that many physicians in the South were disposed to hold themselves aloof from the councils of the Association. He said: "I will not disguise from you, gentlemen, the fact that there are many physicians in the South disposed to hold themselves aloof from your councils. The resolution passed at your meeting in 1866, offering again the hand of fellowship to your Southern brethren, owing to the peculiar condition of the country at that time, and the fact that but little of the medical literature and news of the North circulated with us, met the eyes of but few, and there are still among us those who feel that your hearts are yet steeled against them, and who believe that, notwithstanding some formal declarations to the contrary, most of you, in your private feelings, have not yet been able to rise sufficiently above the prejudices of the past to enable you to receive them in such manner as to make their presence here either agreeable to them or profitable to the Association." * * * * "So far as my observation has extended I am sorry to know these sentiments have prevailed with many, and it is but frankness in me to say so. For myself, and for those whom I represent, I grasp with unaffected pleasure the hands which you have so gracefully and magnanimously offered, and I hope and believe, this sentiment will meet a ready response from all our brethren at the South. *Let us again be united as friends and brothers.*"

We are perhaps unable to appreciate the occasion for all this, but certainly the sentiment is a noble and worthy one. That there has ever been any real alienation in true professional sympathy between the North and South, we are quite unwilling to admit; the leading and intelligent members of the profession, both South and North, indeed, the world over, have always been friends. Was it ever known that the scientific and professional relations of countries were disturbed or interrupted by any political differences which might exist between them? Has it not always been true, that similar objects and aims, thoughts and purposes, beget the purest friendships and truest sympathies?

We have never had any respect for the disloyal sentiments of the South, but always looked upon it with the deepest abhorrence and contempt. The spirit and letter of the rebellion we most heartily opposed, but we could have at all times received any intelligent, faithful medical officer of the Southern army into professional communion. It has never appeared to us that our differences in political opinion could have any influence upon our professional relations. If our friends at the South have ever believed us unfriendly to them as physicians, as honorable and high minded members of the profession, we are sorry they were so ignorant of the true sentiments of our hearts. If they do not desire our company, because we do not agree with their political views, they must long remain separated.

The famous Gardner resolution, voted to be laid indefinitely upon the table, about which so much has been said, now comes up again for review, and is going the rounds of discussion. At this time we have no desire to review the action of the Assoctation, though much might be said in its favor. There is no just ground for construing that vote as opposition to the medical profession of the South, and with that simple declaration we propose to drop, for the present, at least, the Gardner resolution.

The next meeting of the Association is to be held at New Orleans, which, though far distant from us, is yet a very suitable and appropriate place. It is to be hoped that New York and the North generally will be fully represented, and we have no doubt the occasion will be one to unite yet more closely the professional sympathies of the whole country. The idea of Northern aud Southern members of the medical profession not being harmonious and in true fellowship with each other, is absurd, and should no longer deface the pages of professional organs, or the records of Society transactions. We are all brothers, not, it is true, holding the same views upon political questions, and having, very likely, differing opinions upon many subjects, but in all true professional sympathy we are *now* and *always have been* most strongly and firmly united.

Through the efforts of the Permanent Secretary and the committees in aid, physicians can attend the meeting at a comparatively small expense; the season will be favorable for such journey, the relaxation from the monotony of every day life, will be conducive to health, and the facilities for renewing old friendships and of forming new ones, will be unsurpassed. We advise all our readers to embrace the golden opportunity.

